# Use of next generation sequencing to investigate the microbiota of experimentally induced wounds and the effect of bandaging in horses

**DOI:** 10.1371/journal.pone.0206989

**Published:** 2018-11-26

**Authors:** Louis J. Kamus, Christine Theoret, Marcio C. Costa

**Affiliations:** Department of Veterinary Biomedical Sciences, University of Montreal, Saint-Hyacinthe, Quebec, Canada; NYU Langone Medical Center, UNITED STATES

## Abstract

**Objectives:**

To use next generation sequencing to characterize the microbiota of horses during healing of skin wounds in two anatomical locations (body and limb) known to present different healing patterns; and to investigate the impact of bandaging on bacterial communities of skin wounds located on the limbs of horses.

**Methods:**

Full-thickness skin wounds were created on the distal extremity of both thoracic limbs and on one lateral mid-thoracic wall of four healthy horses. Limb wounds were randomly assigned to bandaging or not. A full-thickness sample was collected with a biopsy punch from intact thorax and limb skin (T0) and from the margin of one wound per site (thorax, unbandaged limb, bandaged limb) 1 week (T1) and 2 weeks (T2) postoperatively, and at full healing (T3). Thoracic skin samples obtained from three healthy horses were included in the analysis as controls.

**Results:**

Anatomic location (thorax vs. limb) significantly influenced bacterial composition of equine skin and healing wounds. Fusobacterium and Actinobacillus were strongly associated with limb wounds during the initial phases of healing. Bandaging had a significant impact on the microbiota during the healing process. The skin microbiota after healing was more similar to samples from controls, demonstrating the resilience and stability of the environment.

**Conclusions:**

Equine skin microbiota is a rich and stable environment that is disturbed by wounding, but returns to its previous stage after full healing. Anatomic location significantly influences bacterial composition of the equine skin during wound healing. Bandaging has a significant impact on the skin microbiota of horses during the healing process. Results of this study provide new insight for a better understanding of the contribution of bacteria to wound healing in horses and may facilitate the future development of therapeutic strategies using commensal bacteria.

## Introduction

Wound management is an important component of equine practice because horses’ “flight instinct” predisposes them to injury [1–4]. Trauma to the skin often requires labour-intensive treatment, which generates significant financial commitment, since primary closure is seldom successful and second intention healing is fraught with complications [5]. Healing wounds in horses often become chronic, especially when they are located on the limb, where persistent inflammation is associated with the development of exuberant granulation tissue (EGT) [6].

Many factors may impair wound healing, most notably bacterial infection [5]. In humans, skin contains up to one billion microorganisms/cm^2^, collectively referred to as the microbiota, which provides protection against disease when skin is intact [7]. When injury compromises the skin barrier, microbes can populate sterile tissues leading to bacterial overgrowth and infection, which delay and complicate wound healing. Wounds at the distal extremity of the horse’s limb are highly susceptible to infection as the limb is often contaminated with feces and soil.

Chronic wounds in humans are polymicrobial, with bacterial populations cooperating to promote their survival in a biofilm, thereby perpetuating the chronic nature of the infection [8]. Biofilm is defined as a community of microorganisms attached to a surface, or to each other, who live within a self-synthetized three-dimensional matrix of extracellular polymeric substances [9]. This structure can contain one or more species and enhances the resistance to the host’s immune response and to antimicrobial agents. A few studies reported the presence of biofilm in the wounds of horses [10,11], suggesting that biofilm might impair wound healing also in this species. Jorgensen et al. (2017) detected biofilm in experimental wounds on the limb but not on the body of horses; moreover, biofilm was significantly more prevalent in limb wounds that were bandaged [12]. That study suggested that intact skin microbiota may differ according to anatomic location in horses and that high biofilm burden in limb wounds might relate to proximity to an extreme bacterial load (i.e. ground), and to management practices. Despite the evident importance of the role of bacteria in wound healing, the full skin microbiota has been just recently explored in the horse [13].

Until recently, microbiologists have mostly relied on culture techniques to elucidate the complexity of infections. However, culture techniques detect only organisms that grow readily in laboratory media (5–20% of bacterial species) [14] and bacteria in chronic wound pathogenic biofilms are generally recalcitrant to culture. Moreover, culture of isolates collected using a swab can lead to an overrepresentation of surface bacteria and an underrepresentation of isolates residing deeply within the wound [15]. In horses, the microbiology of intact skin and of wounds has been scantly documented, mostly using conventional techniques such as Gram staining, aerobic and anaerobic culturing, or denaturing gradient gel electrophoresis [10,11,16]. Consequently, information is lacking on the characterization of bacterial populations that occur in association with chronic wounds in horses.

Next generation sequencing (NGS) has been used to characterize the chronic wound microbiota of human patients [17], and to measure the impact of various therapeutic alternatives to standard antimicrobials, for example commensal microbiota [18] or probiotics [19] in an effort to curb antimicrobial resistance. Notably, NGS diagnostics led to a 22.9% faster healing rate compared to culture information [20], consistent with literature reporting that the use of molecular surveys enables detection of bacterial species undetected by culture-based reporting. A better understanding of horse skin microbiota and bacterial populations associated with chronic wound pathogenic biofilms should enable development of next generation therapeutics and may be achieved through the use of novel molecular techniques, such as NGS.

The objectives of this study were to use NGS to investigate the microbiota of a group of horses in two anatomical locations (thoracic wall and distal limb) during the early and late healing phases in experimental full-thickness excisional skin wounds; and to investigate the effects of bandaging on the bacterial communities of skin wounds on the distal limb.

## Materials and methods

### Wound model

Archived samples collected as part of another study and obtained from four healthy mixed breed mares, 5–12 years of age, with no evidence of dermatological disease, wounds or scars, were used in the study. Horses were bought from a local private dealer where they access to *ad libitum* hay and were turned-out outside in a large paddock during the day and kept in stalls overnight. Prior to the study, horses were dewormed and vaccinated (tetanus, encephalitis, influenza and rabies) and allowed an acclimation period of 2 weeks following purchase. Horses were kept in individual box stalls on wood shavings changed daily, and allowed *ad libitum* access to grass hay and water. Each horse was examined daily for signs of discomfort, lameness, and systemic illness; vital parameters, appetite, as well as bandages, were evaluated. The study was conducted in compliance with guidelines for the care and use of laboratory animals as sanctioned by the Canadian Council on Animal Care and approved by the Institutional Animal Care and Use Committee of the University of Montreal (#15-Rech-1811).

Horses were restrained in stocks and sedated (detomidine hydrochloride 0.01mg/kg, butorphanol tartrate 0.04mg/kg, iv), then a square area of 25cm X 25cm on one randomly assigned thoracic wall, and on the surface of both metacarpal regions, was clipped and surgically prepared by scrubbing with 2% chlorhexidine gluconate soap for 5 minutes and rinsing with isopropyl alcohol. Local anesthesia was performed using 2% lidocaine hydrochloride: an inverted L-block was used just below the carpus to desensitize the dorsolateral surface of the cannon while an inverted L-block performed craniodorsally to the wound area desensitized the thoracic wall. Based on a previously established equine wound model [21], with some variations, full-thickness skin wounds were created on the distal extremity of both thoracic limbs (two, 6.25cm^2^ wounds per limb, placed 4cm apart—model of chronic wound healing) and on one lateral mid-thoracic wall (two, 15cm^2^ wounds placed 4cm apart—model of normal wound healing) [22]. All wounds were left to heal by second intention. The wounds on the right or left limb were randomly assigned to bandaging (model of EGT) [23] and the contralateral wound was left uncovered. Bandaged limbs received a traditional half-limb bandage consisting of a low-adherent gauze-like dressing (Melolite, Smith & Nephew) covered by cotton wool roll held in place with an adhesive tertiary layer. Bandages were changed every 2–3 days until 4 weeks post-wounding (estimated time to the start of the remodeling phase), then wounds were left uncovered until full healing. Wounds developing EGT, defined as irregular granulation tissue with many grooves and clefts that protrudes over the margins of the wound, were sharply excised with a sterile scalpel when appraised as grade IV, as described [24]. Wounds on the thoracic wall were left unbandaged for the duration of the study, as per clinical practice. No antimicrobial drugs were given prior to or during the study. Pain was managed with butorphanol tartrate (0.08 mg/kg), as required.

At both anatomical sites, skin removed upon wound creation was kept as the time 0 (T0) sample (normal, intact skin). Following sedation, anesthesia and skin antiseptic preparation as described earlier, wounds to be harvested were gently cleaned with saline-soaked gauze and a full-thickness wound margin sample was collected with an 8mm diameter biopsy punch from one wound per site (body, unbandaged limb, bandaged limb) 1 (T1) and 2 (T2) weeks postoperatively, and at full healing (T3). Furthermore, intact skin samples from the thoracic wall of three healthy horses enrolled in a different project were included in the analysis and designated as controls (CON). Samples from control wounds were collected using the same biopsy protocol, but without prior surgical antiseptic preparation. All samples were snap frozen in liquid nitrogen immediately following collection, and stored at -80°C while awaiting DNA extraction.

### Microbiota evaluation

Total DNA was extracted from each sample using QIAGEN DNeasy Blood & Tissue Kit (Qiagen, Toronto, ON, CA) following manufacturer’s instructions. The V4 region of the bacterial 16S rRNA gene was amplified by PCR using the primers S-D-Bact-0564-a-S-15 and S-D-Bact-0785-b-A-18 [25] in a dual-indexing sequencing strategy: the first PCR consisted of 2 min at 94°C and 33 cycles of 30 sec at 94°C, 30 sec at 58°C and 30 sec at 72°C with a final 7 min at 72°C. Illumina adapters incorporation was carried out at 10 min at 95°C followed by 15 cycles of 15 sec at 95°C, 30 sec at 60°C and 60 sec at 72°C with a final period of 3 min at 72°C. Sequencing was performed using an Illumina MiSeq IEMFile version 4 platform, using a reagent kit V2 (2x250 cycles) at the Genome Quebec Innovation Centre. Sequences are available at the NCBI Sequence Read Archive (SRA) under accession number SRP163273.

Bioinformatic analysis was carried using the software mothur [26]. Good quality reads were clustered in operation taxonomic units (OTUs) at the genus level (>94% similarity) and classified according to the Ribosomal Database Project (RDP) databank. The number of observed genera, Chao1 richness estimator, Shannon and Simpson’s index were used for characterization of alfa diversity. Beta diversity (comparison between communities) was addressed by the Jaccard and the Yue and Clayton indices, to compare respectively community membership (that considers the different bacterial taxa) and structure (that considers the different taxa and how they are distributed within the community). The similarity between communities’ membership and structure was compared using the Principal Coordinate Analysis (PCoA) and the Unweighted Pair Group Method with Arithmetic Mean (UPGMA) algorithm, visualized by tree diagrams (dendrograms).

### Statistical analysis

Alfa diversity indices were compared over time using repeated measures ANOVA considering the time of sampling and the anatomical location of the wound (thorax or limb) as variables. Alfa diversity indices were then compared between anatomical locations and managements (bandaged versus unbandaged) using the Student’s t-test considering each time of sampling individually. Linear Discriminant Analysis Effective Size (LEfSe) [27] was used to find differences in the relative abundances to detect meaningful biological differences between sampling times, anatomical sites and between managements. Community membership and structure were compared with the Parsimony (t test) and the analysis of molecular variance (AMOVA) tests.

## Results

**Fig 1:**
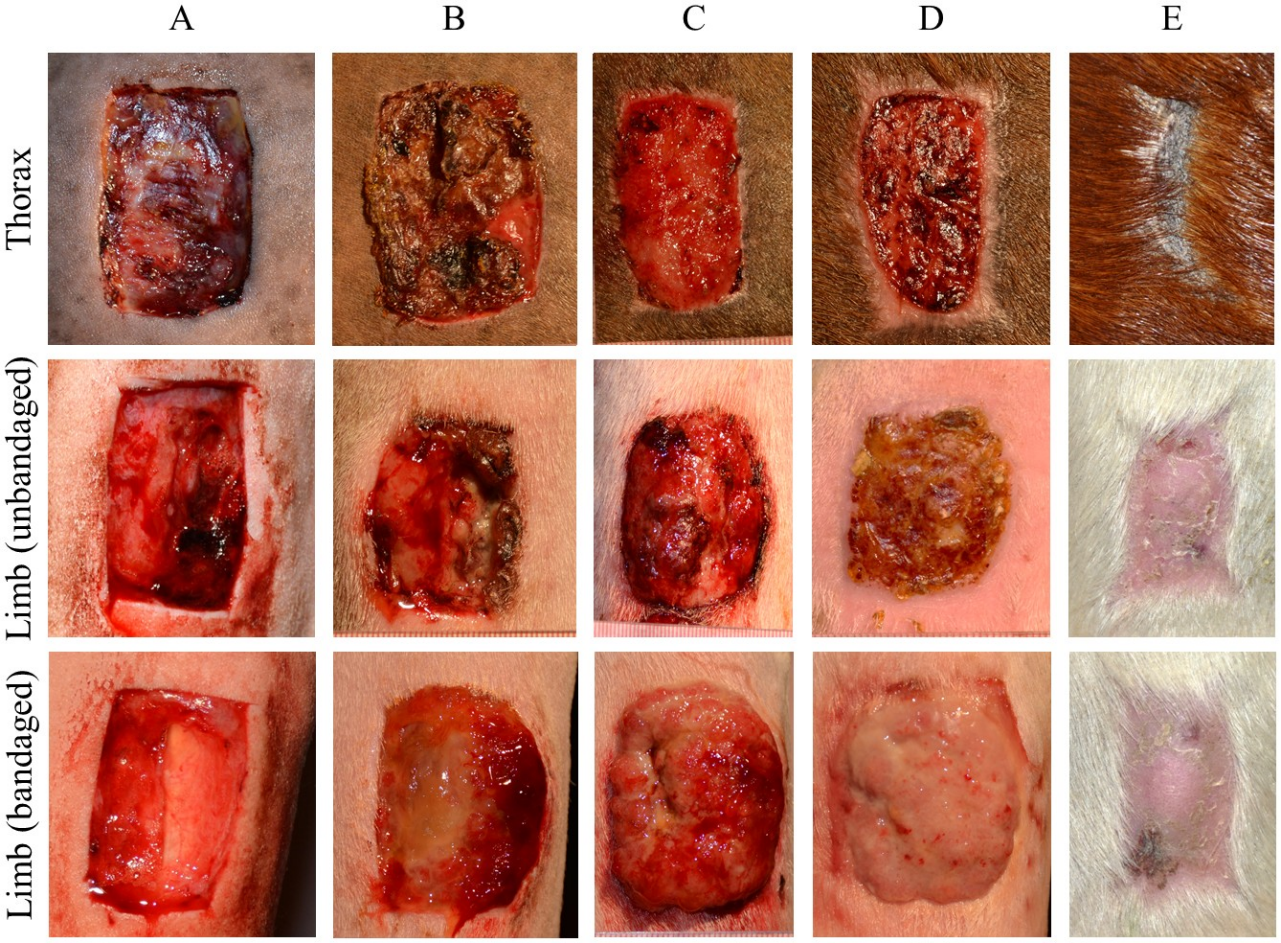
Healing of experimentally induced wounds of one single horse at the different body sites. A: 24h, B: 7 days, C: 14 days, D: 21 days, E: cicatrix.

No signs of discomfort nor abnormalities in vital parameters recorded during physical examination were observed throughout the study period, and therefore analgesia was not required. As expected of this model, limb wounds took longer to heal than did body wounds: average time to full healing for unbandaged limb wounds was 83 days (SD 2.58 days) and that for body wounds was 62.5 days (SD 2.52 days). All bandaged, but no unbandaged limb wounds developed EGT. Time for healing of bandaged limb wounds was not recorded, but since all developed EGT, it took approximately 7 days longer than unbandaged wounds. The different healing phases in each anatomical location are presented in Fig 1.

### Characterization of equine skin microbiota

A total of 4,321,050 good quality sequences were used for the final analysis (mean, 88,185 per sample; SD, 38,986). The sample providing the lowest number of reads (6,848) was used as a cut-off for subsampling the other samples to decrease bias caused by non-uniform samples. Using this cut-off, the average coverage was 99.64% (SD, 0.30), indicating that the analysis could detect almost all genera estimated to be present on the skin of horses.

**Fig 2:**
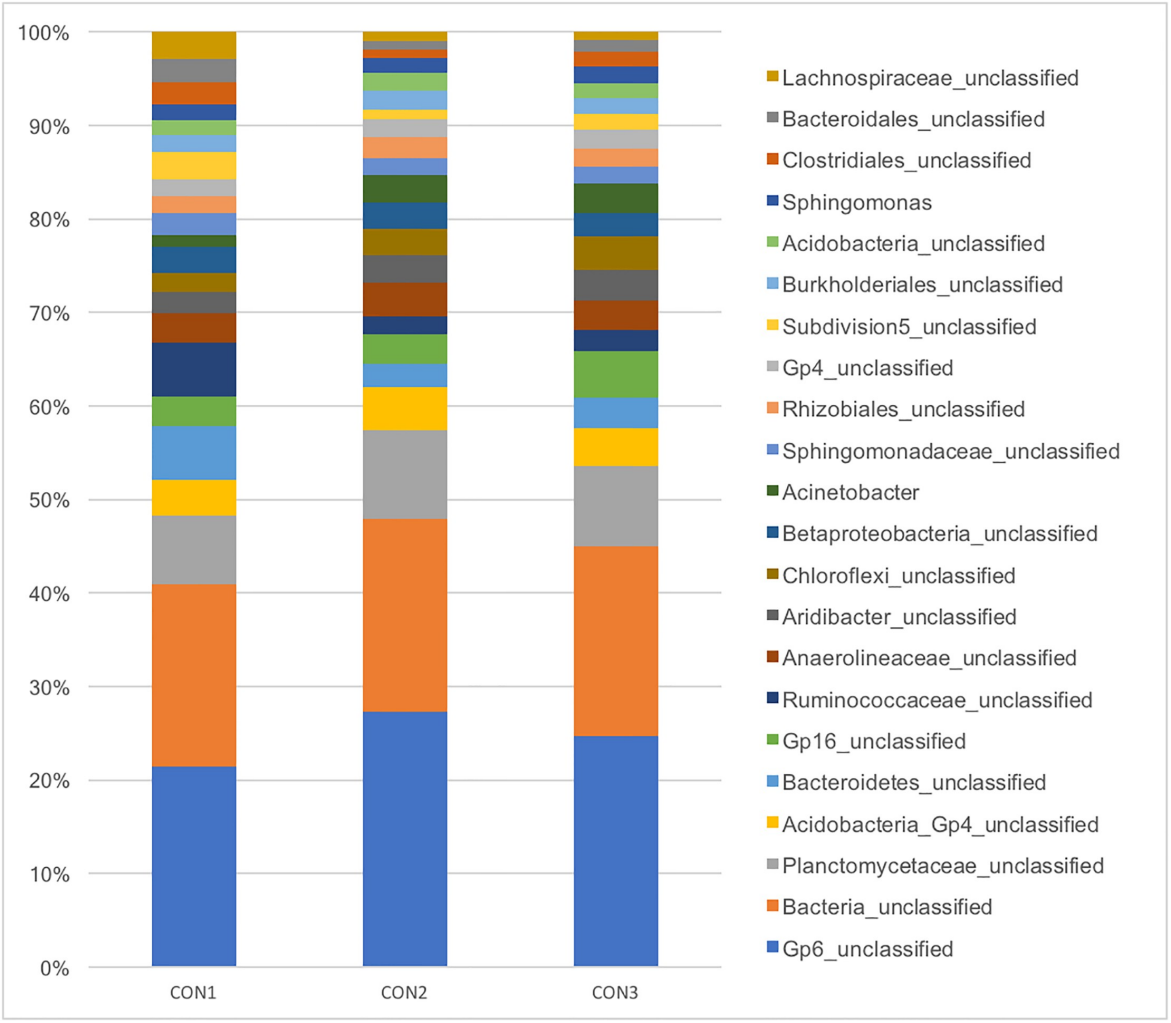
Relative abundance of the main genera bacterial genera (>1%) found in skin biopsies of three healthy horses.

Virtually all of the most abundant genera (>1%) found in the skin of healthy horses from the control group were unclassified at the genus level and most of them belonged to the Acidobacteria phylum. Abundances of the main genera (>1%) present on skin of control horses are presented in Fig 2.

**Fig 3:**
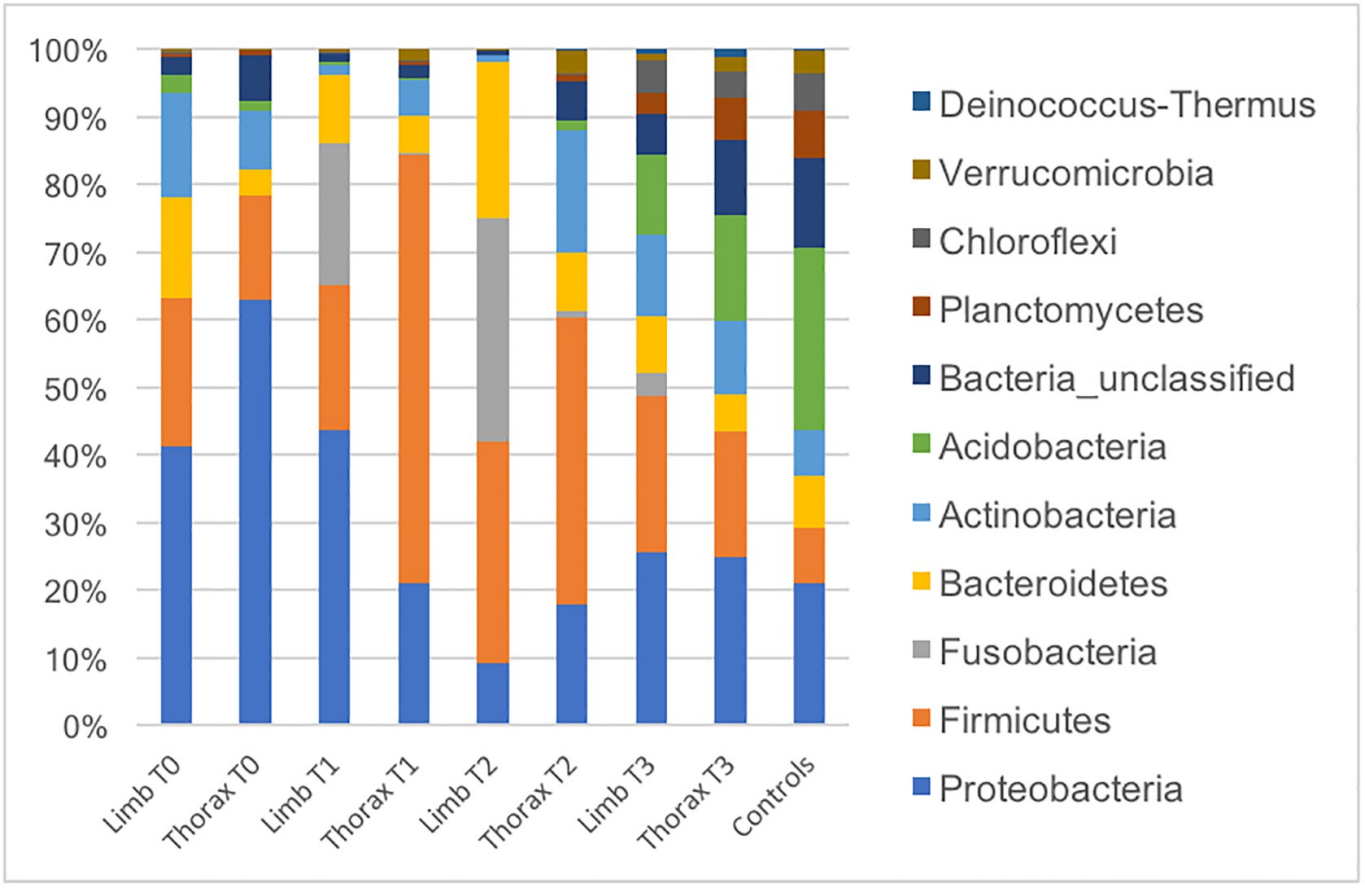
Relative abundances of main bacterial genera found at different body sites (thorax and limbs) during wound healing in horses. T0: after surgical scrubbing; T1: 1-week post wounding; T2: 2-weeks post wounding; T3: full healing. Bandaged limb wound group excluded.

Changes in microbiota composition over the healing process and the comparison between anatomical sites are presented in Fig 3. S1 and S2 Figs show the relative abundance of the main phyla and genera, respectively, for each individual included in the study. It is interesting to note the inter-individual variability of the composition of the main populations in each sample, especially at the genus level.

Comparison of the two anatomical locations (thorax vs. unbandaged limbs) performed by the LefSe analysis revealed that Fusobacterium and Actinobacillus were strongly associated (LDA score >4) with limb wounds at T1 and T2. Other significant associations can be observed in S3 and S4 Figs.

**Fig 4:**
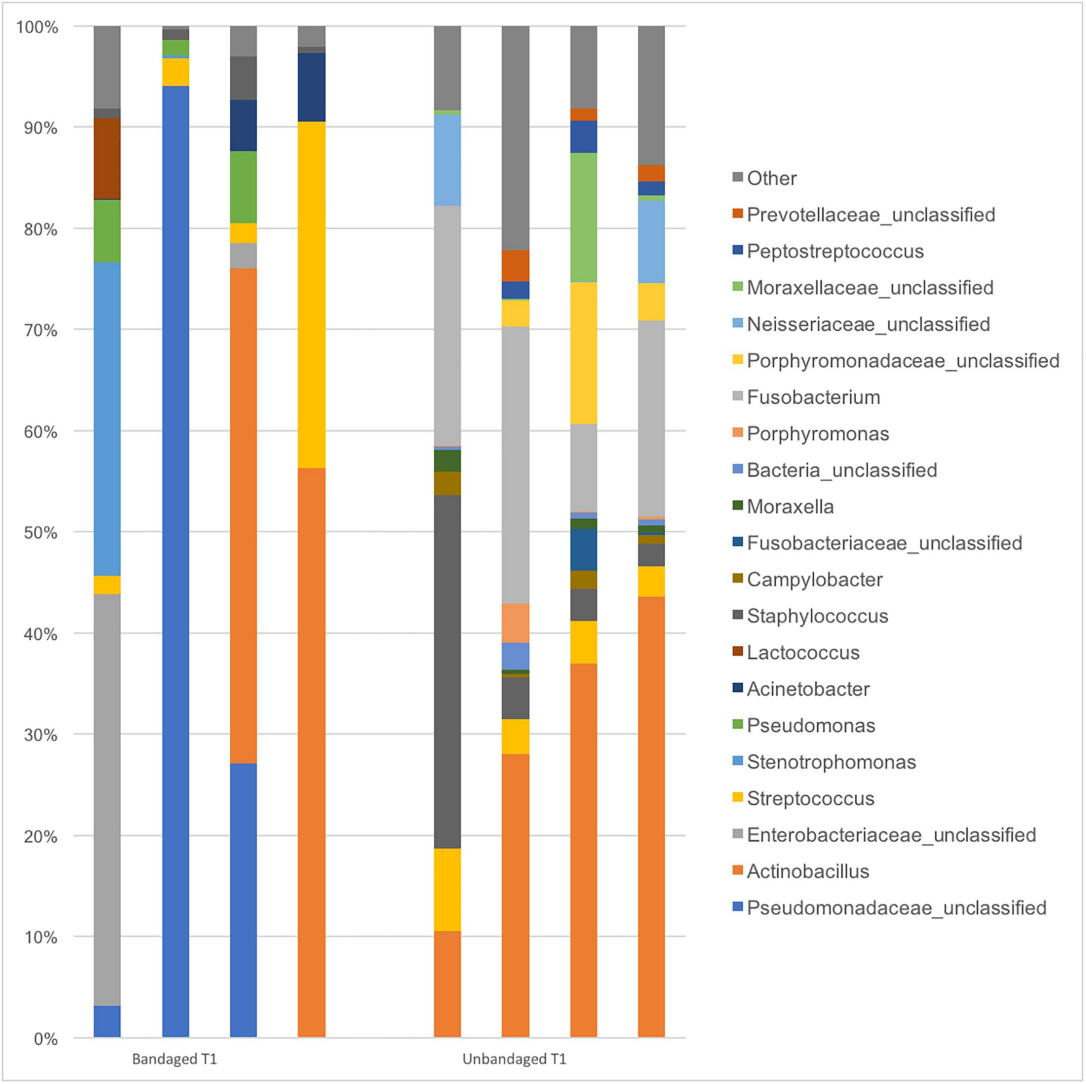
Relative abundances of main bacterial genera found in the limb of horses 1-week post wounding (T1) under two different managements (bandaged and unbandaged).

Comparison of the two wound management approaches (bandaged vs. unbandaged limb wounds) revealed greater variability in communities present in wounds from bandaged limbs (Fig 4). Fusobacterium, unclassified Neissereaceae and Omonadaceae were associated with unbandaged limb wounds at T1 and unclassified Prevotellaceae and Parvimonas at T2 (LefSe analysis). The full list of bacteria associated with limb wounds left unbandaged is presented in S5 and S6 Figs.

### Alfa diversity

Mean values and standard deviation (SD) of alfa diversity indices within each sampling time and results of statistical analysis comparing anatomical sites and management are presented in Table 1.

**Table 1:** Mean values, standard deviation (SD) and P values of statistical comparison of alfa diversity indices found in the skin of horses at different moments of wound healing. T0: after surgical scrubbing; T1: 1-week post wounding; T2: 2-weeks post wounding; T3: full healing.

	**# genera**	**Chao**	**Simpson**	**Shannon**
Limb—unbandaged				
T0	50 (22)	60 (23)	8 (4)	2 (1)
T1	108 (34)	138 (45)	5 (1)	2 (0)
T2	60 (30)	79 (35)	4 (2)	2 (1)
T3	187 (90)	218 (109)	25 (16)	4 (1)
Limb—bandaged				
T1	23 (7)	33 (7)	3 (1)	1 (1)
T2	31 (17)	44 (23)	2 (1)	1 (0)
T3	132 (69)	160 (71)	11 (13)	3 (2)
Thorax				
T0	55 (37)	66 (47)	6 (5)	2 (1)
T1	105 (67)	126 (73)	10 (14)	2 (1)
T2	108 (35)	126 (36)	16 (5)	3 (0)
T3	263 (42)	315 (47)	18 (11)	4 (1)
Controls	307 (10)	384 (12)	19 (2)	4 (0)
**Statistics (P values)**	**# genera**	**Chao**	**Simpson**	**Shannon**
Limb x thorax*				
T0	0.408	0.409	0.372	0.335
T1	0.465	0.396	0.241	0.365
T2	0.405	0.055	**0.003**	**0.020**
T3	0.089	0.763	0.248	0.234
Bandaged x Unbandaged				
T1	**0.001**	**0.002**	**0.008**	0.057
T2	0.072	0.073	0.054	**0.025**
T3	0.187	0.205	0.084	0.057
Thorax x Controls				
T0	**<0.001**	**<0.001**	**0.004**	**0.014**
T1	**0.002**	**<0.001**	0.163	0.053
T2	**<0.001**	**<0.001**	0.201	**0.026**
T3	0.070	**0.030**	0.440	0.218
Overtime changes				
Limb—unbandaged	0.225	0.208	0.146	0.376
Limb—bandaged	0.163	0.131	0.571	0.091
Thorax	0.105	0.111	0.152	0.384

* unbandaged limb samples only

values in bold font indicate comparisons that are statistically significant

Main findings include more diverse communities in thoracic wounds compared to limb wounds at T2. Unbandaged limb wounds had a richer and more diverse community compared to bandaged limbs at T1. The same trend was observed at T2, but no statistical significance was achieved, probably due to small sample size and substantial inter-animal variability observed among samples from bandaged limb wounds.

### Beta diversity

**Fig 5:**
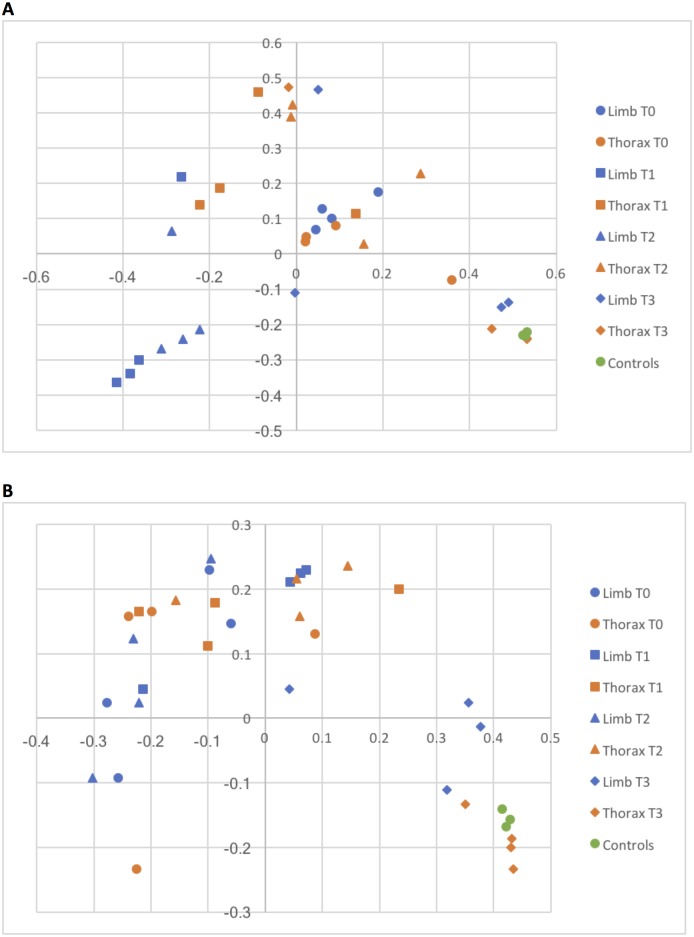
PCoA of membership (A) and structure (B) of bacterial communities collected during the healing process from the thoracic and limb wounds of horses. Only unbandaged limb wound samples were included in this graphical representation. T0: after surgical scrubbing; T1: 1-week post wounding; T2: 2-weeks post wounding; T3: full healing.

Similarities between communities’ membership (that considers only which genera were present in each sample) and structure (that accounts for the relative abundance of each genus) are represented in Fig 5. Noteworthy, there was a great spatial separation between limb and thoracic wound samples at T1 and T2, which was confirmed by statistical analysis (Table 2).

**Table 2:** P values obtained from statistical analyses comparing community membership and structure of bacterial communities found through wound healing in horses.

	Membership	Structure
**Limb x Thorax—overall**		
Parsimony	0.054	**0.015**
AMOVA	**0.032**	**0.002**
**Control x Limb—overall**		
Parsimony	**0.007**	**0.009**
AMOVA	**<0.001**	**<0.001**
**Control x Thorax—overall**		
Parsimony	**0.014**	**0.018**
AMOVA	**0.008**	**0.007**
**Bandage x Unbandage T1**		
Parsimony	0.097	0.498
AMOVA	**0.027**	0.075
**Unbandage limbs T1 x T3**		
Parsimony	**0.014**	0.107
AMOVA	**0.002**	**0.002**
**Unbandage limbs T2xT3**		
Parsimony	**0.020**	0.325
AMOVA	**0.001**	**0.004**

values in bold font indicate comparisons that are statistically significant

There were no statistically significant differences between bacterial communities in samples from limb and thoracic wounds after healing (T3). In fact, microbiota similarity of those samples can be observed by the clustering in the PCoA (Fig 5), in which T3 samples appear together with samples from controls with normal skin, suggesting a stable microbiota that returns to a “normal” state once the healing process is complete.

**Fig 6:**
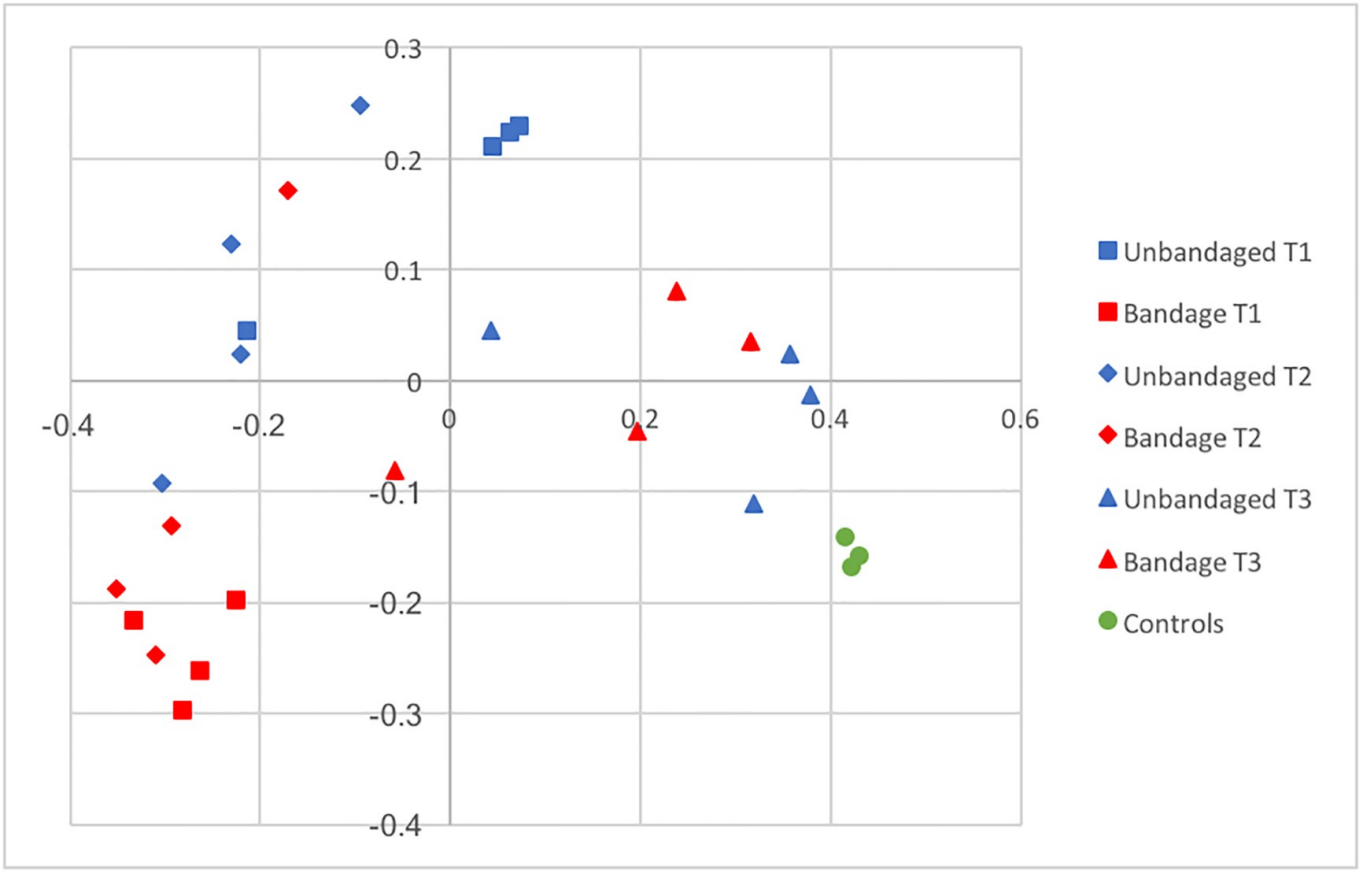
PCoA representing bacterial community membership similarity during the healing process in limb wounds according to management (bandaged and unbandaged) and over time. T1: 1-week post wounding; T2: 2-weeks post wounding; T3: full healing.

**Fig 7:**
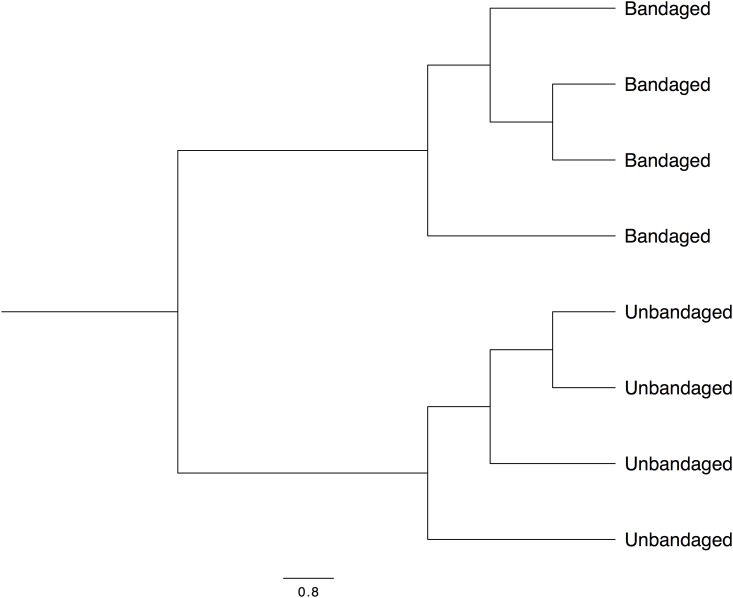
Dendrogram representing similarity of bacterial membership of microbiota present in limb of horses under two managements (bandaged and unbandaged) 1 week after wounding (T1).

The comparison between management approaches revealed differences in membership, that can be clearly visualized in Figs 6 and 7. However, statistical significance was not consistent across all tests applied (Table 2) probably because of the great variability in communities present between bandaged individuals.

## Discussion

### Skin microbiota of healthy horses

The great similarity in the skin microbiota of the three control horses (Figs 2 and 5) was suggestive that this is a stable environment that is consistent across individuals, as it has been shown in other species [28] and in horses [13]. Acidobacteria comprised the vast majority of bacteria present on the skin of control horses. This difficult-to-grow bacterium has been reported to be of low abundance as identified by NGS in the skin of humans and mice [29] and was associated with occurrence of psoriasis in humans [30]. This demonstrates that the skin microbiota of horses is unique and extrapolation of data from humans or laboratory animals may not be accurate for the equine species. Further studies designed to better characterize Acidobacteria living in the equine skin at the species level and its role as a commensal organism are warranted. Although Ross et al. (2018) reported *Corynebacterium* spp. as the most common bacterial genus found in horses, this elegant study focuses on the comparison between different species but does not provide much detail on the full composition of the equine skin microbiota [13]. More studies investigating this environment are required.

The fact that most bacteria found in this study were unclassified at the genus level along with 20% unclassified bacteria at the phylum level, reinforces the need of further efforts to investigate this unexplored environment. Considering the strict bioinformatics methods adopted for this analysis, it is unlikely that those unclassified DNA sequences are the consequence of sequencing errors [31]. Furthermore, the same pipeline and database have been used to successfully classify up to 97% of genera present in other environments [32,33]. Therefore, this finding may truly reflect the presence of a high number of unknown bacteria in the skin of horses, which might be of special importance for the development of new therapies.

### Temporal changes during healing

The present study provides evidence that bacterial communities change in a predictable fashion during the healing process. These preliminary observations are an important step towards a better understanding of the role of bacteria in wound healing in horses and might be the basis for the exploration of innovative prophylactic approaches.

Despite empirical use of cutaneous probiotics in horses, to date, there is no strong evidence supporting the effectiveness of such products. It is well established that commensal bacteria play a major role in mucosal wound healing [34], but the efficacy of probiotics in cutaneous wound healing is controversial, mainly because well controlled studies evaluating this topic are scarce. Nevertheless, there is increasing evidence that commensal bacteria may accelerate healing by regulating inflammation through stimulation of regulatory T cells and skin dendritic cells [35,36].

The dynamics of bacterial communities during the healing process can be observed from Figs 3 and 5. Interestingly, microbial communities present in fully healed cutaneous wounds (T3) clustered with those of intact skin from control samples. Together, this evidence suggests that the skin microbiota of horses is markedly altered during healing, likely in relation to a local inflammatory process [37], but returns to a baseline state that resembles the microbiota of normal, intact skin. Unfortunately, intact skin sampled for this project had been surgically prepared, therefore precluding further inferences regarding microbial dynamics within the same individual.

### Influence of anatomic location

It has been hypothesized that bacterial contamination of the equine limb skin due to proximity to the ground is responsible for an increased inflammatory response, which in turn could be the cause of EGT, a condition that develops frequently and almost exclusively in wounds located on the limb [12]. The present study demonstrated differences in bacterial composition of wounds between body sites as well as higher bacterial diversity in the thoracic location, as it has been demonstrated in humans [38]. Diversity is usually associated with more stable and resilient communities while decreased diversity is present in the face of dysbiosis (microbial imbalances) [39,40] however, the consequences of such observations in equine wound healing remain purely speculative at this point.

While this study showed differences in microbial communities between body sites, association of specific organisms with certain aspects of healing (i.e.: healing time, presence of EGT) exceeded the scope of this study, and further investigation comparing different body sites without previous surgical preparation, or using clinical cases, is necessary. Although it is tempting to speculate that differences in microbiota between anatomic locations could be the cause of chronicity in the limb wounds of horses [41], the design of the present study does not allow inference of a cause-consequence relationship (i.e.: role of bacteria by modulating the immune system vs. microbiota changes caused by inflammation). Nevertheless, *Fusobacterium* and *Actinobacillus* spp. were strongly associated with limb wounds during the initial phases of healing, and could potentially exacerbate the inflammatory response, as it has been demonstrated in other tissues [42]. This finding may direct future studies to elucidate the role of those organisms in wound chronicity in the horse.

Interestingly, differences in microbiota between body and limb wounds at earlier times of healing (T1, T2) disappeared once wounds were healed (i.e. as these differences resolve, so does the chronic inflammation that is hindering healing of the limb wounds). Indeed, samples obtained from healed wounds (T3), had very similar communities regardless of anatomic location, which were also clustering with the microbiota found in the normal skin of control samples (Figs 3 and 5).

### Management

The differences in alfa and beta diversity indices observed between bandaged and unbandaged limb wounds indicate that it is possible to manipulate the skin microbiota during the healing process. In addition to differences in community membership, the richer and more diverse community in unbandaged limb wounds at T1 (Tables 1 and 2) suggests that bandaging alters environmental conditions possibly via an effect on temperature, moisture, debris, oxygen tension and decreasing interaction with the ground microbiota. The potential impact of topical wound treatments and wound-care products on a wound’s microbiota is a nascent and promising field of research.

Although differences in membership are clearly visualized in Figs 6 and 7, statistical significance was not consistent across all tests (Table 2) probably because of the great variability in communities present in bandaged wounds and the small sample size (n = 4) available for the study. Finally, it appears that the effect of bandaging on wound microbiota may cause long-term changes, since several genera present after full healing (T3) were associated only with wounds that had been left unbandaged (S5 Fig). However, the consequences associated with such bacterial changes remain to be investigated before formulating management recommendations.

### Limitations

Some limitations of this study deserve attention. Firstly, only four horses were included in this study and results should not be extrapolated to a broader population, especially considering that this experimental model uses antisepsis of the skin, simulating a surgical procedure. Therefore, further studies evaluating contaminated wounds obtained from clinical cases are necessary.

A higher number of animals would also decrease inter-individual variation, as observed in S2 Fig. Nevertheless, for this type of studies it is essential that the environment be as standardized as possible, so that other factors will not influence the results (such as including other barns), and the similarities in beta diversity observed in each treatment and time are suggestive that those changes were indeed caused by healing.

Sampling timing was designed in order to cover the early phases of healing: hemostasis and acute inflammation (T1), fibroplasia at the beginning of the remodeling phase (T2), and wound closure (T3). More sampling times would have probably allowed a better evaluation of the microbiota dynamic through healing.

It is important to note that the DNA sequencing technology used here is limited to taxonomic classification at higher levels (i.e. family) and therefore suitable for visualization of bacterial shifts over time or differences in communities between groups, but not to identify specific organisms. Also, these methods are sensitive to detect DNA, which should be considered, for example, when interpreting the presence of bacterial DNA at T0 (after surgical preparation): the presence of bacterial DNA does not mean the bacteria were live or viable.

Finally, this is a descriptive study not intended to infer any cause-and-effect relationship with our clinical findings.

## Conclusions

Equine skin microbiota is a rich and stable environment that is disturbed by wounding, but returns to its previous stage after full healing. Anatomic location significantly influences bacterial composition of the equine skin during wound healing. Bandaging has a significant impact on the skin microbiota of horses during the healing process. Results of this study provide new insight for a better understanding of the contribution of bacteria to wound healing in horses and may facilitate the future development of therapeutic strategies using commensal bacteria.

## Supporting information

S1 Fig Relative abundances of main bacterial phylum found in limb wounds of four horses under two different managements: Bandaged and unbandaged. T0: after surgical scrubbing; T1: 1-week post wounding; T2: 2-weeks post wounding; T3: full healing. Bandaged limb wound group excluded.
(TIFF)

S2 Fig Relative abundances of main bacterial genera found in limb wounds of four horses under two different managements: Bandaged and unbandaged. T0: after surgical scrubbing; T1: 1-week post wounding; T2: 2-weeks post wounding; T3: full healing. Bandaged limb wound group excluded.
(TIFF)

S3 Fig LefSe analysis indicating meaningful associations of bacterial taxa at different body sites (thorax and limbs) 1 week after wounding. (TIFF)

S4 Fig LefSe analysis indicating meaningful associations of bacterial taxa at different body sites (thorax and limbs) 2 weeks after wounding. (TIFF)

S5 Fig LefSe analysis indicating meaningful associations of bacterial taxa at different body sites (thorax and limbs) after wound healing. (TIFF)

S6 Fig LefSe analysis indicating meaningful associations of bacterial taxa in two different managements (bandaged and unbandaged) in horses 2 weeks after limb wounding. (TIFF)
