# Supplementary figures and images for: Use of next generation sequencing to investigate the microbiota of experimentally induced wounds and the effect of bandaging in horses
Source: PLoS One. 2018 Nov 26;13(11):e0206989. doi: 10.1371/journal.pone.0206989 (PMC6261015; doi:10.1371/journal.pone.0206989)

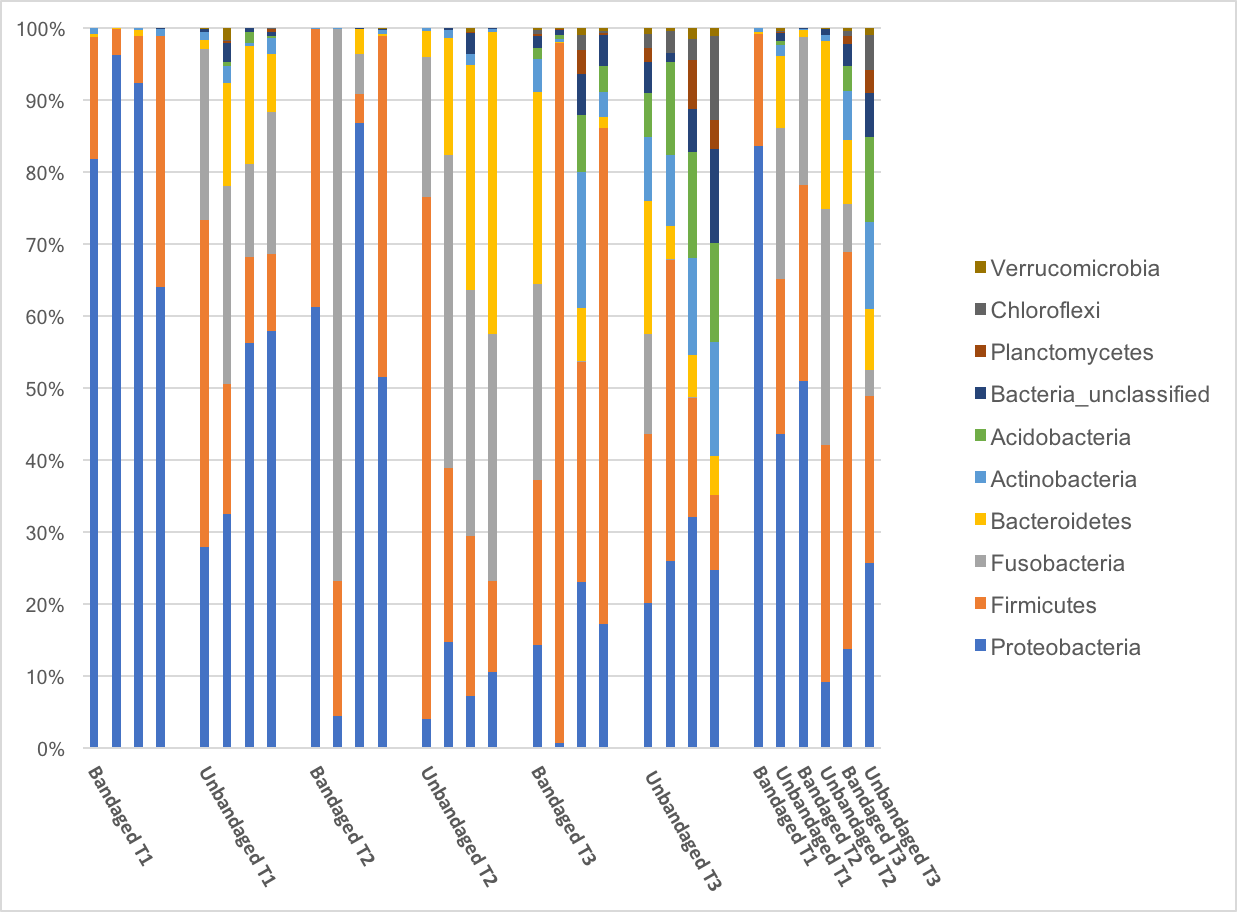

Supplement: S1 Fig — T0: after surgical scrubbing; T1: 1-week post wounding; T2: 2-weeks post wounding; T3: full healing. Bandaged limb wound group excluded. (TIFF) [file pone.0206989.s001.tiff]

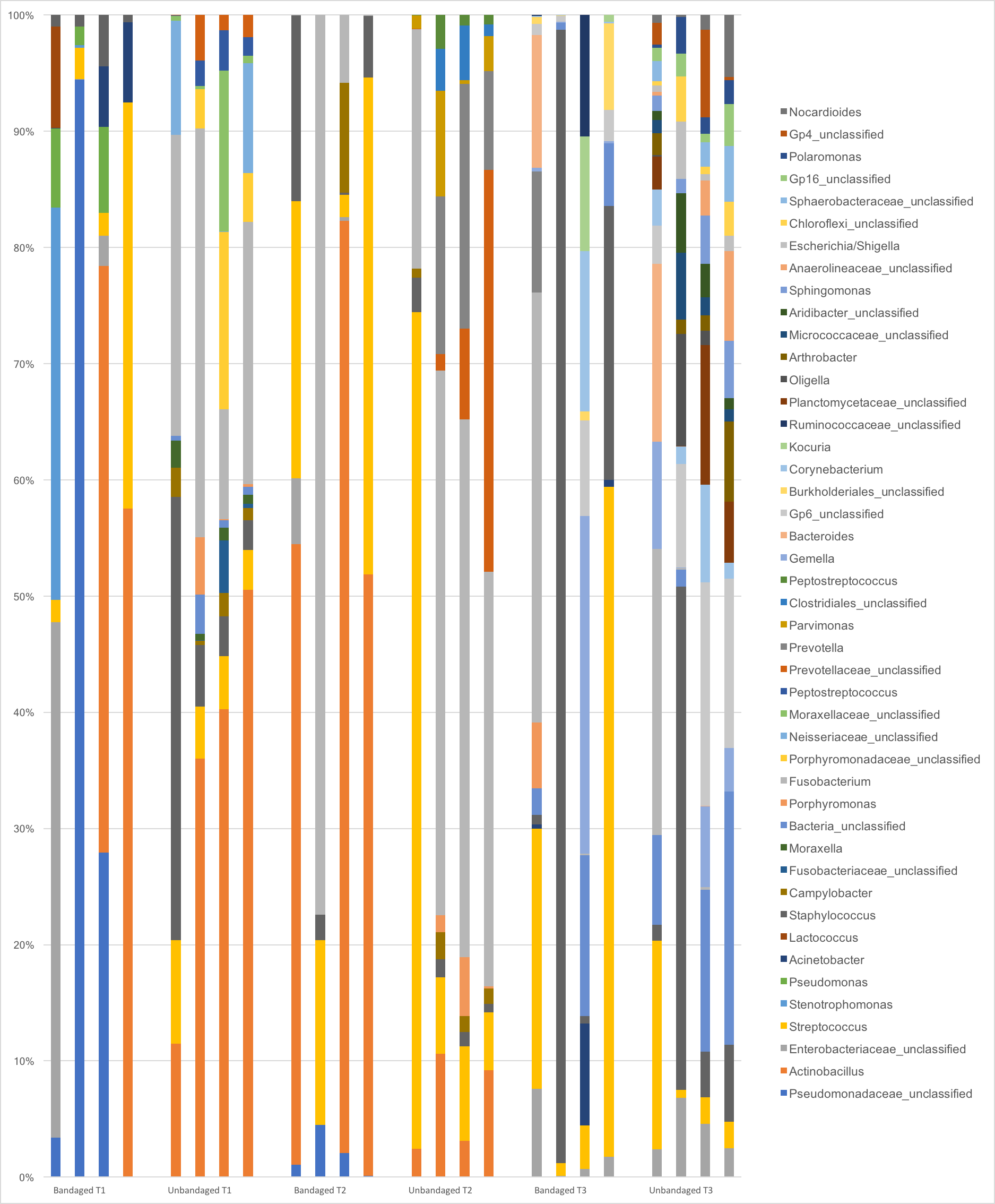

Supplement: S2 Fig — T0: after surgical scrubbing; T1: 1-week post wounding; T2: 2-weeks post wounding; T3: full healing. Bandaged limb wound group excluded. (TIFF) [file pone.0206989.s002.tiff]

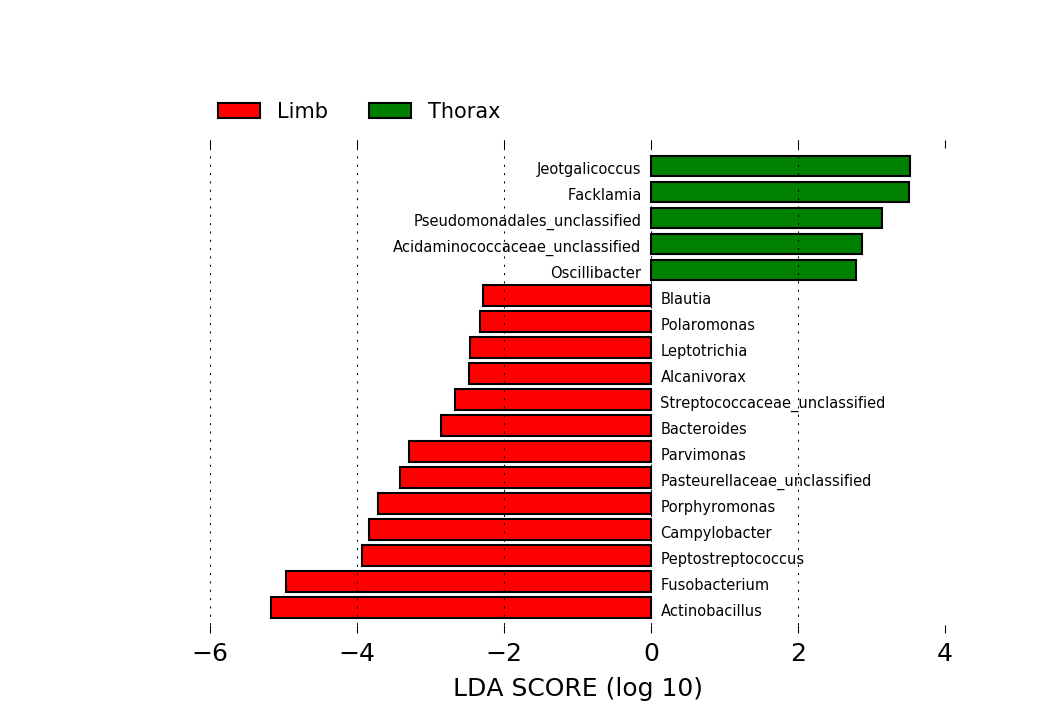

Supplement: S3 Fig — (TIFF) [file pone.0206989.s003.tiff]

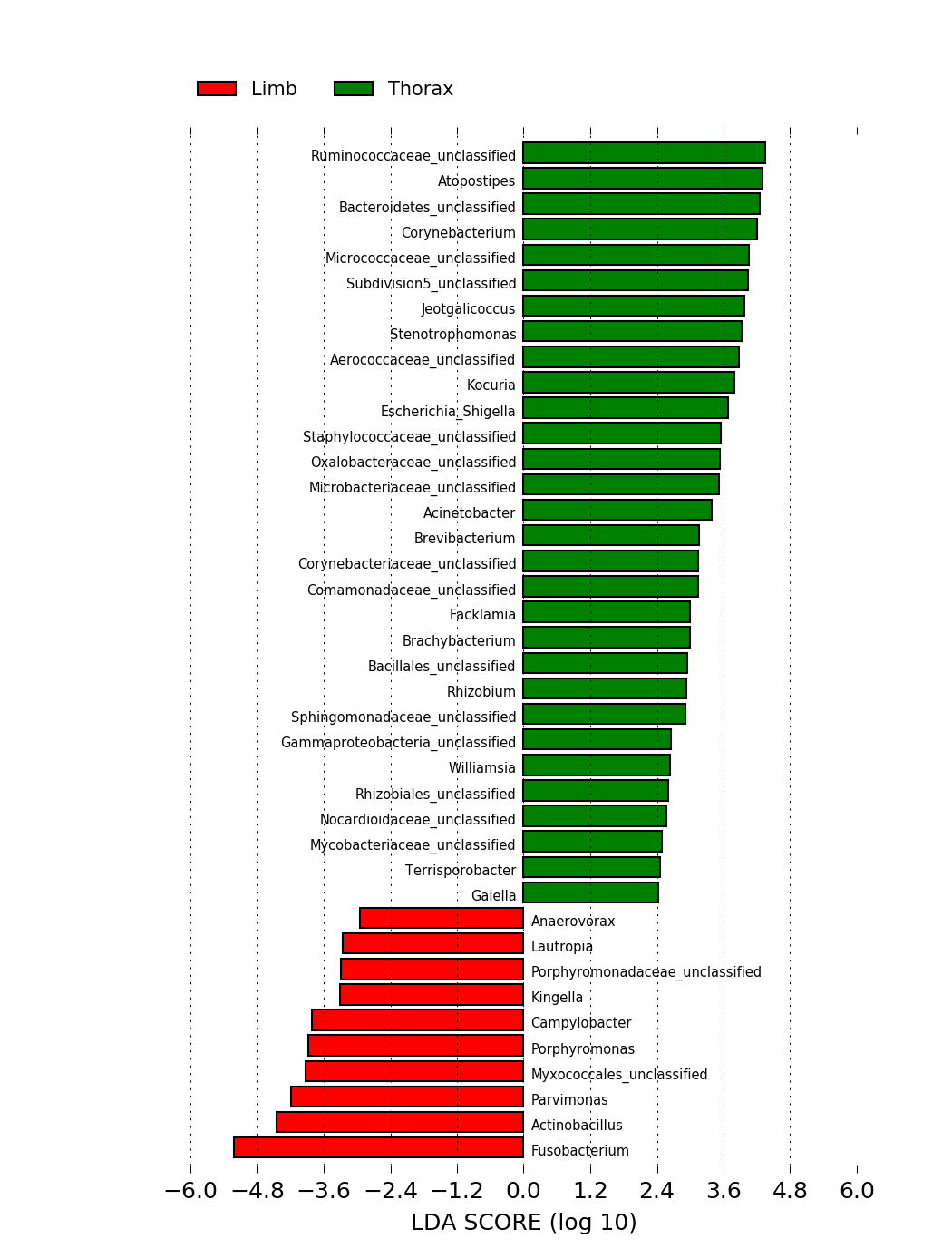

Supplement: S4 Fig — (TIFF) [file pone.0206989.s004.tiff]

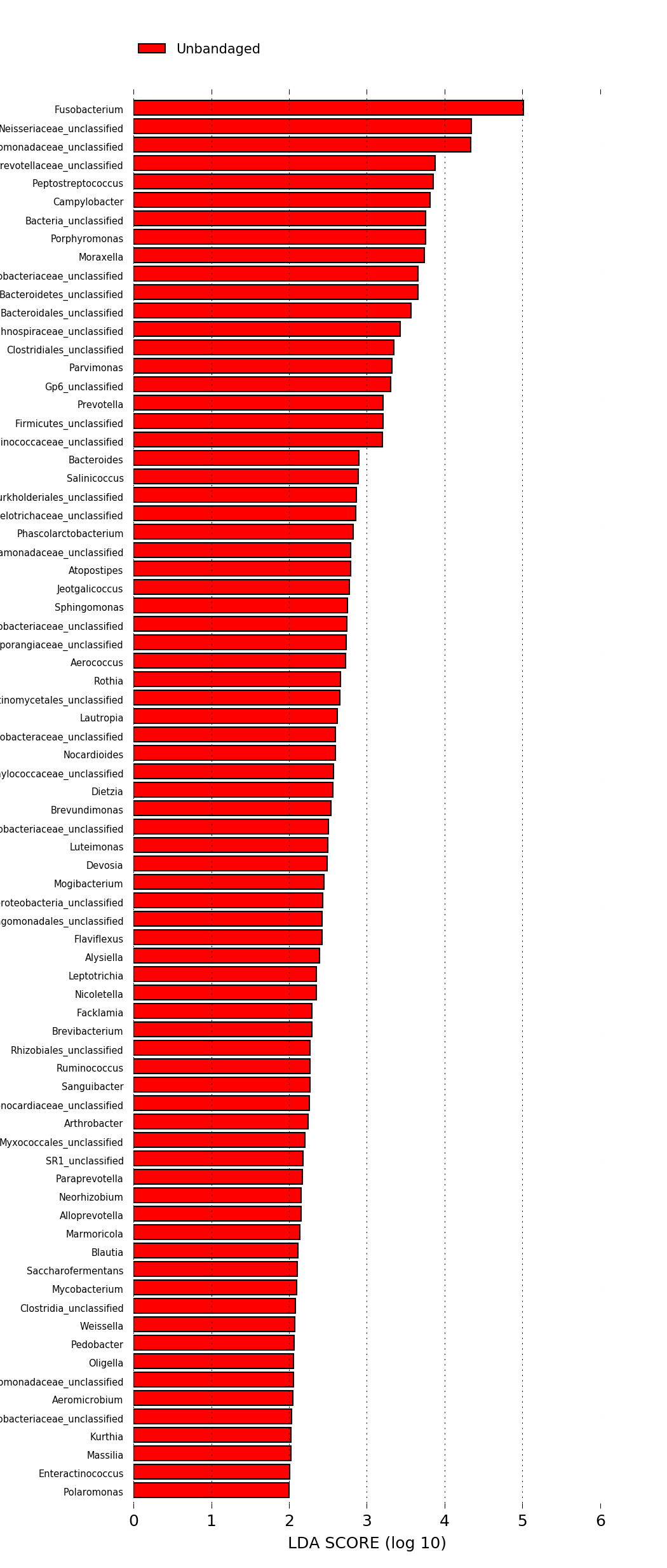

Supplement: S5 Fig — (TIFF) [file pone.0206989.s005.tiff]

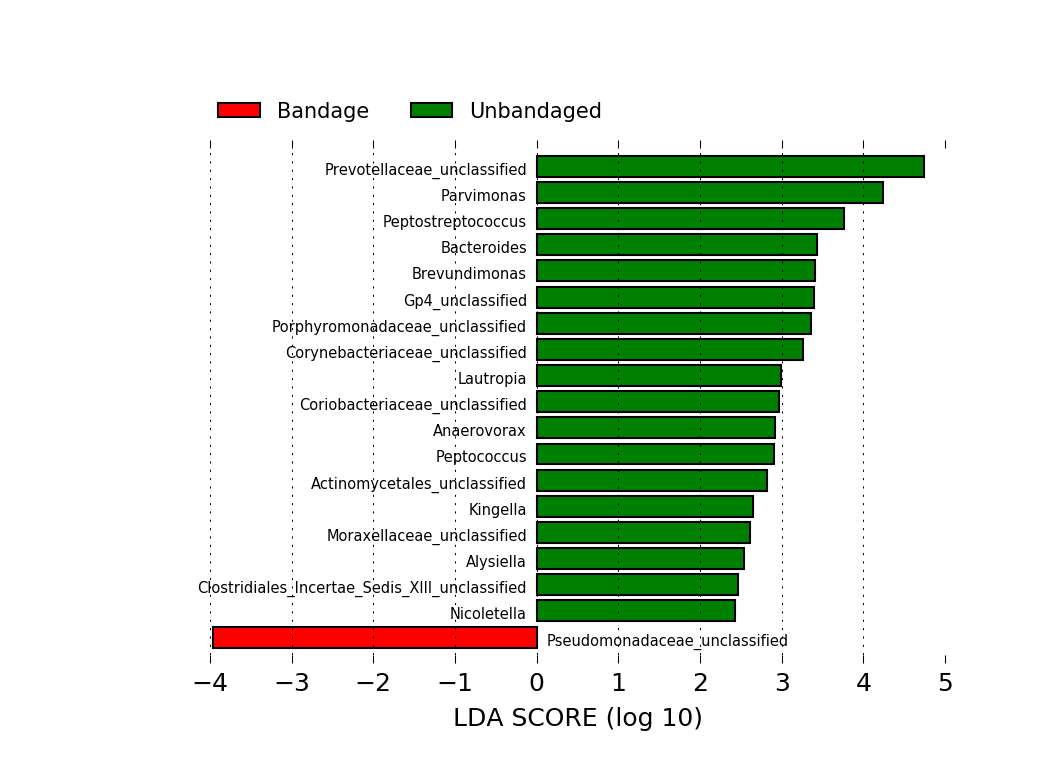

Supplement: S6 Fig — (TIFF) [file pone.0206989.s006.tiff]
